# Integrating biological knowledge into variable selection: an empirical Bayes approach with an application in cancer biology

**DOI:** 10.1186/1471-2105-13-94

**Published:** 2012-05-11

**Authors:** Steven M Hill, Richard M Neve, Nora Bayani, Wen-Lin Kuo, Safiyyah Ziyad, Paul T Spellman, Joe W Gray, Sach Mukherjee

**Affiliations:** 1 The Netherlands Cancer Institute, 1066 CX Amsterdam, The Netherlands; 2 Centre for Complexity Science, University of Warwick, Coventry CV4 7AL, UK; 3 Department of Statistics, University of Warwick, Coventry CV4 7AL, UK; 4 Genentech Inc., San Francisco, CA 94080; 5 Life Sciences Division, Lawrence Berkeley National Laboratory, Berkeley, CA 94720; 6 Center for Spatial Systems Biomedicine, Oregon Health & Science University, Portland, OR 97239

## Abstract

**Background:**

An important question in the analysis of biochemical data is that of identifying subsets of molecular variables that may jointly influence a biological response. Statistical variable selection methods have been widely used for this purpose. In many settings, it may be important to incorporate ancillary biological information concerning the variables of interest. Pathway and network maps are one example of a source of such information. However, although ancillary information is increasingly available, it is not always clear how it should be used nor how it should be weighted in relation to primary data.

**Results:**

We put forward an approach in which biological knowledge is incorporated using informative prior distributions over variable subsets, with prior information selected and weighted in an automated, objective manner using an empirical Bayes formulation. We employ continuous, linear models with interaction terms and exploit biochemically-motivated sparsity constraints to permit exact inference. We show an example of priors for pathway- and network-based information and illustrate our proposed method on both synthetic response data and by an application to cancer drug response data. Comparisons are also made to alternative Bayesian and frequentist penalised-likelihood methods for incorporating network-based information.

**Conclusions:**

The empirical Bayes method proposed here can aid prior elicitation for Bayesian variable selection studies and help to guard against mis-specification of priors. Empirical Bayes, together with the proposed pathway-based priors, results in an approach with a competitive variable selection performance. In addition, the overall procedure is fast, deterministic, and has very few user-set parameters, yet is capable of capturing interplay between molecular players. The approach presented is general and readily applicable in any setting with multiple sources of biological prior knowledge.

## Background

Ongoing advancements and cost reductions in biochemical technology are enabling acquisition of ever richer datasets. In many settings, in both basic biology and medical studies, it may be important to model the relationship between assayed molecular entities, such as genes, proteins or metabolites, and a biological response of interest.

Molecular players may act in concert to influence biological response: this has motivated a need for multivariate methods capable of modelling such joint activity. When sample sizes are small-to-moderate, as is often the case in molecular studies, robust modelling of joint influences becomes especially challenging. However, often it is likely that only a small number of players are critical in influencing the response of interest. Then, the challenge is to identify appropriate variable subsets.

Statistical variable selection methods have been widely used in the bioinformatics domain to discover subsets of influential molecular predictors. Both penalised likelihood and Bayesian approaches have been used in a diverse range of applications [1-6].

Bayesian approaches can facilitate the integration of ancillary information regarding variables under study through prior probability distributions. Ongoing development of online tools and databases have meant that such information is widely available, and depending on context, may include networks and pathway maps, public gene expression datasets, molecular interaction databases, ontologies and so on. However, while the idea of incorporating such information into variable selection has a clear appeal, it is not always obvious what information should be included nor how it should be weighted. Indeed, many existing Bayesian variable selection approaches do not attempt integrative analyses exploiting such information and instead employ standard priors that do not specify preferences for particular variables, but may, for example, encode a preference for sparse models [4,7]. Several Bayesian variable selection studies have put forward simple approaches for incorporating prior knowledge by independently assigning each variable a prior probability of being included in the model [1,6,8,9]. However, subjectively setting such hyperparameters for each variable may be difficult. Furthermore, prior independence may be a questionable assumption, since molecular variables are unlikely to influence a response independently of one another.

We develop a variable selection procedure in which an empirical Bayes approach is used to objectively select between a choice of informative priors incorporating ancillary information (‘biologically informative priors’) and also to objectively weight the contribution of the prior to the overall analysis. The work presented here is motivated by questions concerning the relationship between signalling proteins and drug response in human cancers. In the protein signalling setting (as also in gene regulation) there is now much information available, both in the literature and in diverse online resources, concerning relevant pathways and networks. We therefore develop pathway- and network-based informative priors for this setting, applying the methods proposed to automatically select and weight the prior and thence carry out variable selection.

The relationship between response and predictors is modelled using a continuous, linear model with interaction terms. In this way we avoid data discretization (which can lose information) yet retain the ability to capture combinatorial interplay. We take advantage of biochemically-motivated sparsity constraints to permit exact inference, thereby avoiding the need for approximate approaches such as Markov chain Monte Carlo (MCMC). This enables the calculation of exact probability scores over which variables are likely to be influential. The overall procedure is computationally fast: empirical Bayes analysis and subsequent calculation of posterior (inclusion) probabilities for 52 predictors via full model averaging required only 10 minutes (in MATLAB R2010a on a standard single-core personal computer; code freely available, together with simulation scripts, at http://go.warwick.ac.uk/stevenhill/IBKVS). Moreover, the overall procedure we put forward is simple from the user perspective, requiring very few user-set parameters or MCMC convergence diagnostics.

The remainder of the paper is organised as follows. We begin below by defining notation and reviewing Bayesian variable selection. We then describe methods, including empirical Bayes analysis to objectively select and weight biologically informative prior information, pathway-based informative priors and exact inference. We illustrate our method on published single cell proteomic data [10] and on proteomic data and drug response from ongoing work in breast cancer. We also compare the proposed approach to alternative methods. We conclude with a discussion of our results, the merits and shortcomings of our work, and highlight directions for further work.

### Notation

Let $Y={\left(Y_{1},\ldots ,Y_{n}\right)}^{T}\in R^{n}$ be a vector of response values and $X_{i}\in R^{p}$ for $i\in \left\{1,\ldots ,n\right\}$ be corresponding *p*-dimensional candidate predictors. **X**_*i *_ forms row *i* of the *n *×* p *predictor matrix **X**.

Let $\gamma ={\left(\gamma_{1},\ldots ,\gamma_{p}\right)}^{T}\in {\left\{0,1\right\}}^{p}$ be a binary vector and $\left|\gamma \right|=\underset{j}{\sum }\gamma_{j}$ be the number of non-zeros in *γ*. Then **X**_*γ*_ is the $n\times \left|\gamma \right|$ matrix obtained by removing from **X** those columns *j* for which *γ*_*j *_= 0. Similarly, for a vector $a=\left(a_{1},\ldots ,a_{p}\right)$, **a**_*γ*_ is obtained from **a** by removing components *a*_*j *_for which *γ*_*j *_= 0.

### Bayesian variable selection

#### Bayesian linear model

Consider the classical linear model **Y **=** X*β*** + *ε*, where $\beta ={\left(\beta_{1},\ldots ,\beta_{p}\right)}^{T}$ are regression coefficients and $\varepsilon ∼N\left(0,\sigma^{2}I\right)$, where N denotes a Normal distrbution. In some settings it makes sense to assume that some of the regression coefficients can be set to zero, thereby removing the corresponding predictors from the model. Variable selection addresses the question of which subset of predictors best models the response. An inclusion indicator vector $\gamma \in {\left\{0,1\right\}}^{p}$ specifies which regression coefficients vanish. That is, predictor *j* is included in the model if and only if *γ*_*j *_= 1. We use *γ * to denote both the inclusion indicator vector and the model it specifies. Given model *γ* we have the reduced linear model

(1) $$Y=X_{\gamma }\beta_{\gamma }+\varepsilon .$$

We are interested in the posterior distribution over models *P*(*γ* | **Y**,**X**). From Bayes’ rule we have

(2) $$P(\gamma \;|\;Y,X)\propto p(Y\;|\;\gamma ,X_{\gamma })P(\gamma )$$

where *p*(**Y** | *γ*,**X**_*γ*_) is the marginal likelihood. *P*(*γ*) is a prior over models (the ‘model prior’), and is the main focus of the present paper. The marginal likelihood is obtained by integrating out the regression parameters *β*_*γ*_ and variance parameter *σ*^2^ and thereby automatically penalises complex models with many parameters. This penalisation occurs because a more complex model has a larger parameter space. This means that for more complex models, the integral that defines the marginal likelihood is over a greater number of dimensions, with prior mass spread over a larger space. This in turn results in a lower marginal likelihood score.

#### Model selection and model averaging

The posterior distribution over models *P*(*γ* | **YX**) can be used to find a single, *maximum a posteriori* (MAP) model, $\hat{\gamma }={\text{argmax}}_{\gamma }P\left(\gamma \;|\;Y,X\right)$. However, considering a single ‘best’ model may be misleading, especially in the small-sample setting where the posterior distribution is likely to be diffuse with several similarly high-scoring models. Model averaging [11,12] can ameliorate such effects by averaging over the entire space of models to calculate posterior inclusion probabilities for each individual predictor,

(3) $$P(\gamma_{j}=1\;|\;Y,X)=\underset{\gamma :\gamma_{j}=1}{\sum }P(\gamma \;|\;Y,X).$$

These inclusion probabilities are a measure of the importance of each individual predictor in determining the response.

Evaluating the summation in Equation 3 requires enumerating the entire posterior over models *P*(*γ* | **YX**). The model space Γ can be vast ( $\left|\Gamma \right|=2^{p}$) even for moderate values of *p* . Thus, Markov chain Monte Carlo [13] is often used to sample from the posterior over models thereby providing asymptotically valid estimates of the inclusion probabilities [4]. As outlined in Methods below, we instead calculate exact inclusion probabilities; an approach rendered computationally viable through restricting the size of model space Γ. Justifications for such a restriction and advantages and disadvantages of an exact approach are provided in the Discussion below.

#### Model prior

Calculating the posterior distribution over models (2) requires specifying a prior over Γ, *P*(*γ*). A common choice of prior assumes that the *a priori* inclusion probabilities are independent and have Bernoulli distributed marginal distributions *P*(*γ*_*j*_) with success parameter *Π*. This hyperparameter may be a user-defined constant or may itself have a Beta prior [9,14]. In the former case, small values are often chosen to promote parsimonious models [1].

These priors provide no information regarding specific predictors and do not utilise domain knowledge. Employing predictor dependent hyperparameters *Π*_*j *_enables incorporation of prior knowledge that some predictors are more important than others. However, utilising such a prior may be difficult in practice due to the many hyperparameters that must be subjectively specified. We note also in this formulation, prior inclusion probabilities are still independent.

## Methods

We now describe the Bayesian variable selection method used in the present work. We describe in turn, an extended linear model including interactions between predictors, exact computation of posterior inclusion probabilities, biologically informative model priors and empirical Bayes learning of associated hyperparameters.

### Bayesian linear model with interaction terms

We extend the classical linear model in Equation 1 above to enable combinatorial relationships between predictors and response to be captured. Given model *γ*, response *Y*_*i*_ depends in a non-linear fashion on the included predictors **X**_*iγ *_whilst remaining linear in the regression parameters. In particular, the mean for *Y*_*i*_ is a linear combination of included predictors and all possible products of included predictors. For example, if $\left|\gamma \right|=2$ with *γ*_3_ =* γ*_5_ = 1, we have *Y*_*i *_=** X**_*iγ*_*β*_*γ*_ + *αX*_*i*3_*X*_*i*5_ + *ε*_*i*_. We extend the $n\times \left|\gamma \right|$ predictor matrix **X**_*γ*_ and regression coefficient vector ***β***_*γ *_to include the interaction terms and coefficients respectively, and we denote the extended versions by ${\bar{X}}_{\gamma }$ and ${\bar{\beta }}_{\gamma }$. All columns in ${\bar{X}}_{\gamma }$ are standardised to have zero mean and unit variance.

The likelihood now takes the form

(4) $$p(Y\;|\;\gamma ,{\bar{X}}_{\gamma },{\bar{\beta }}_{\gamma },\sigma^{2})∼N\left({\bar{X}}_{\gamma }{\bar{\beta }}_{\gamma },\sigma^{2}I\right).$$

We choose hierarchical parameter priors following Smith and Kohn [15] and Nott and Green [14], taking the prior for ${\bar{\beta }}_{\gamma }$ given *γ* and *σ*^2^ to be Normal

(5) $$p({\bar{\beta }}_{\gamma }\;|\;\gamma ,{\bar{X}}_{\gamma },\sigma^{2})∼N\left(\text{0},n\sigma^{2}{\left({\bar{X}}_{\gamma }^{T}{\bar{X}}_{\gamma }\right)}^{-1}\right)$$

and the prior for *σ*^2^ to be $p(\sigma^{2})\propto \sigma^{-2}$. Integrating out the parameters results in the following closed form marginal likelihood,

(6) $$\begin{array}{l} p(Y\;|\;\gamma ,X_{\gamma })\propto {\left(1+n\right)}^{-\frac{2^{\left|\gamma \right|}-1}{2}}\\ \;\times {\left(Y^{T}Y-\frac{n}{n+1}Y^{T}{\bar{X}}_{\gamma }{\left({{\bar{X}}_{\gamma }}^{T}{\bar{X}}_{\gamma }\right)}^{-1}{{\bar{X}}_{\gamma }}^{T}Y\right)}^{-\frac{n}{2}}. \end{array}$$

We note that, in contrast to the widely-used normal inverse-gamma prior [8,16], this formulation has no free hyperparameters and enjoys attractive invariance properties under rescaling [17].

### Exact posterior inclusion probabilities

We enforce a restriction on the number of predictors that are allowed to be included in the model. That is, we only allow *γ* with $\left|\gamma \right|\leq d_{\text{max}}$ for some $d_{\text{max}}\in N$. Thus, instead of being exponential in *p*, the model space Γ has polynomial size of order $p^{d_{\text{max}}}$, thereby allowing explicit calculation of posterior inclusion probabilities via Equation 3. We take *d*_*max *_= 4, giving $\left|\Gamma \right|=294,204$ for the *p *= 52 predictors in the cancer drug response application below; the original size of Γ was of order 10^15^.

### Biologically informative model priors

We now turn our attention to the model prior *P*(*γ*). In many molecular biology settings, there is much valuable information available which may be used to construct biologically informative model priors. This could be network and pathway structures, providing information on relationships between predictors, or information from publicly available datasets. However, it may not be obvious precisely *how* such information should be used and it is usually possible to encode several different, apparently plausible priors. We are therefore interested in investigating the question of how to choose between such priors.

Suppose we have *M* priors to choose from, with each prior, indexed by $m\in \left\{1,\ldots ,M\right\}$, encoded by a function $f_{m}:{\left\{0,1\right\}}^{p}\to R$ which scores a proposed model *γ * according to the prior information. Following Mukherjee *et al.*[4], we take the overall prior to be of the following form,

(7) $$P(\gamma \;|\;m,\lambda )\propto \text{exp}\left\{\lambda f_{m}(\gamma )\right\}$$

where *m* is a hyperparameter (the ‘source parameter’) that selects amongst priors and *λ* is a hyperparameter controlling the overall strength of the prior.

**Figure 1:**
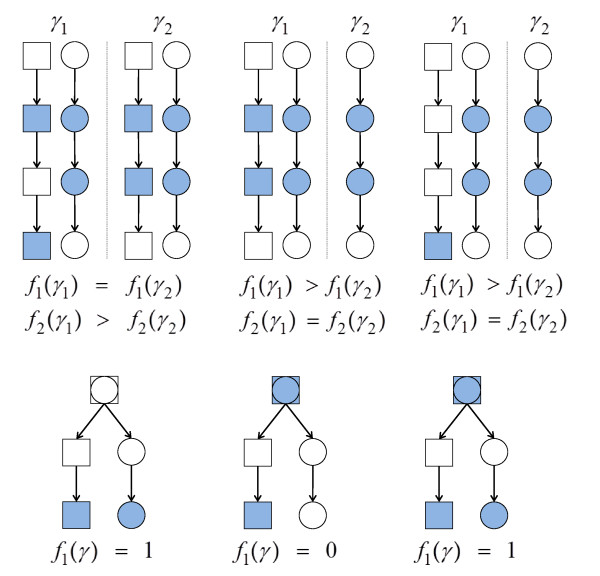
**Properties of pathway-based priors.** Priors are encoded by functions *f*_1_(*γ*) (number of pathways) and *f*_2_(*γ*) (intra-pathway distance). Shaded components are contained in model *γ * and shapes represent different pathways. Top row: Comparisons of the scoring functions. Top left - *γ*_1_ has larger intra-pathway distance than *γ*_2_; Top middle - distance is agnostic to number of pathways; Top right - addition of a singleton has no effect on distance. Bottom row: The root component in each network is in both pathways. However, *f*_1_(*γ*) is defined so as to avoid double counting.

We consider two simple pathway-based priors, capturing information regarding number of pathways and intra-pathway distances via functions *f*_1_ and *f*_2_ respectively. Below we proceed to give details for each, making use of the following notation. We let ${Ε}_{k}\subseteq \left\{1,\ldots ,p\right\}$ denote the set of proteins contained in pathway *k*, $k\in \left\{1,\ldots ,K\right\}$, and we let ${Ε}_{k}^{\gamma }=\gamma \cap {Ε}_{k}$ be the set of proteins that are both in model *γ * and in pathway *k*. We note that a protein is both allowed to be a member of more than one pathway or to not be a member of any pathways. If there is no prior information available, the pathway-based priors reduce to a flat prior over model space. Figure 1 illustrates properties of the two prior components.

#### Number of pathways (*f*_1_)

The first pathway-based feature encodes the notion that predictors that are influential in determining response may belong to a small number of pathways or, in contrast, may be spread across many pathways. We encode such beliefs by a function *f*_1_(*γ*) which counts the number of pathways represented in a model *γ*. Specifically, $f_{1}(\gamma )=\max(0,K_{\gamma }-1)$ where *K*_*γ*_ is the pathway count given by $\underset{S}{\min}\left|S\right|$ for $S\subseteq \left\{1,\ldots ,K\right\}$ satisfying

(8) $$\overset{K}{\underset{k=1}{⋃}}{Ε}_{k}^{\gamma }=\underset{k\in S}{⋃}{Ε}_{k}^{\gamma }.$$

This definition prevents the empty model being *a priori* most probable and avoids double counting (proteins that are members of multiple pathways are considered to be a member of only one pathway for the purpose of calculating *f*_1_(*γ*), and this single pathway is selected to minimise the pathway count; see Figure 1). If the strength parameter *λ* is negative, the prior increasingly penalises models as number of pathways increases, whereas a positive value results in a prior that prefers models representing many pathways.

#### Intra-pathway distance (*f*_2_)

The second feature we consider is that variables which jointly influence the response may either be close to each other in a network sense, or may in fact be far apart in the network. This is done by a function *f*_2_(*γ*) which gives the average distance between pairs of proteins that are both in *γ * and in the same pathway. Specifically, the distance between two proteins *j*_1_ and *j*_2_, denoted *d*(*j*_1_,*j*_2_), is the number of edges in the shortest (undirected) path between them. Then, we define $f_{2}(\gamma )=\max(0,D_{\gamma }-1)$ where *D*_*γ*_ is the average of all *d*(*j*_1_,*j*_2_) with $j_{1},j_{2}\in {Ε}_{k}^{\gamma }$ for some *k*. In order for the distance to be defined for any two proteins in a pathway, we assume that the network topology for a pathway consists of a single connected component (in the undirected sense). We term a protein included in *γ* as a *singleton* if there are no other included proteins in the same pathway (i.e. protein *j* is a singleton if ${Ε}_{k}^{\gamma }=\{j\}$ for some *k*). For models that only contain singletons or the empty model we set *D*_*γ *_= 0. The function *f*_2_(*γ*) defined in this way satisfies a number of natural desiderata. It is agnostic to |γ| and to the pathway count *K*_*γ*_(see Figure 1). Also, it avoids double counting (the distance between each protein pair contributes to *f*_2_(*γ*) once only) and is indifferent between models including only singletons and models with the smallest possible average distance of *D*_*γ *_= 1. A negative strength parameter *λ * results in a prior that penalises larger intra-pathway distances, whilst a positive value encourages larger distances.

### Empirical Bayes

We set the prior source parameter *m* and strength parameter *λ* in an objective manner using empirical Bayes [18]. Specifically, we maximise the following marginal likelihood,

(9) $$\begin{array}{lll} p(Y\;|\;X,m,\lambda ) & =E{\left[p(Y\;|\;\gamma ,X_{\gamma })\right]}_{P(\gamma \;|\;m,\lambda )}\; & \;\\ & =\underset{\gamma }{\sum }p(Y\;|\;\gamma ,X_{\gamma })P(\gamma \;|\;m,\lambda ).\; \end{array}$$

For a given choice of hyperparameters, the marginal likelihood can be calculated exactly by exploiting the model space restriction described above. The score is calculated for varying hyperparameters and those resulting in the largest score are used for variable selection.

### Prediction

Given already observed data **X**,**Y**, we can predict the expected value of new response *Y*^*′ *^ from new predictor data **X**^*′ *^ by model averaging:

(10) $$E\left[Y^{\prime }\;|\;X^{\prime },X,Y\right]=\underset{\gamma }{\sum }E\left[Y^{\prime }\;|\;X^{\prime },X,Y,\gamma \right]P(\gamma \;|\;Y,X)$$

with

(11) $$E\left[Y^{\prime }\;|\;X^{\prime },X,Y,\gamma \right]=\frac{n}{n+1}{\bar{X}}_{\gamma }^{\prime }{\left({{\bar{X}}_{\gamma }}^{T}{\bar{X}}_{\gamma }\right)}^{-1}{{\bar{X}}_{\gamma }}^{T}Y$$

and the model posterior *P*(*γ* | **Y**,**X**) calculated via Equations 2, 6 and 7.

## Results

We first show an application of our proposed approach to synthetic response data generated from a published study of cell signalling, and then further illustrate the approach with an analysis of proteomic data and drug response from breast cancers.

### Synthetic response data

In ongoing studies, such as that presented below, truly objective performance comparisons may be challenging, since we usually do not know which molecules are truly influential in driving biological response. At the same time, in fully synthetic data it can be difficult to mimic realistic correlations between variables within a pathway or across a network. For this reason, we empirically assessed the methods proposed using published single-cell, phospho-proteomic data [10] with responses generated from that data. This preserved pathway-related correlation structure between predictors but permitted objective assessment. The dataset consists of 11 proteins and *n*_*tot *_= 853 samples. (The complete dataset from [10] contains data obtained under nine different conditions, corresponding to different interventions. Here, we use the baseline dataset which contains 853 samples).

**Figure 2:**
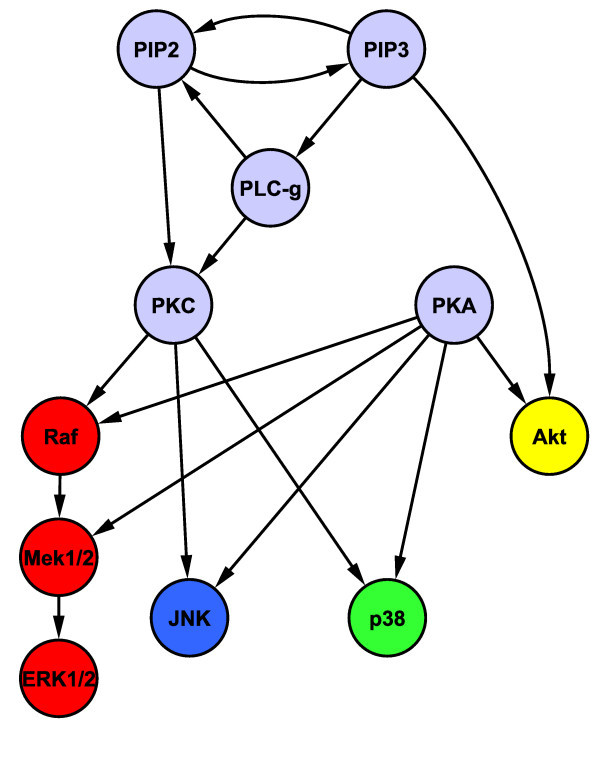
**Protein network and pathway structure for biologically informative priors in the synthetic response data study.** Responses were generated from published phospho-proteomic data [10] consisting of 11 proteins and 853 samples (baseline data only). Network structure shown here is based on that given in Sachs *et al.*[10] and reflects current knowledge of signalling interactions. Proteins were divided into four pathways, denoted by node colours red, blue, green and yellow. The grey nodes are each members of all four pathways.

Figure 2 shows a network and pathway structure for the 11 proteins for use with the biologically informative priors of the type described above. The network structure was taken from Sachs *et al.*[10] and reflects current knowledge of signalling interactions. The proteins were assigned into four pathways.

**Figure 3:**
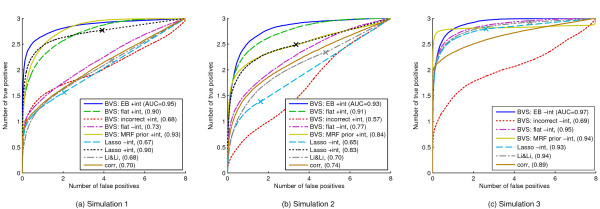
**Synthetic response data, average ROC curves.** Number of true positives plotted against number of false positives for Simulations 1, 2 and 3. Proteomic data from Sachs *et al.*[10] were used to create response data with true underlying model known to favour a particular prior: Simulaton 1 - distance prior with positive *λ*; Simulations 2 and 3 - either distance prior or number of pathways prior with negative *λ*. Legend - ‘BVS’: Bayesian variable selection; ‘+int’: linear model with interaction terms; ‘-int’: linear model without interaction terms; ‘EB’: empirical Bayes used to select and weight pathway-based priors automatically; ‘flat’: flat prior; ‘incorrect’: wrong prior with respect to true, underlying protein set (see main text for details); ‘MRF prior’: Markov random field prior [19]; ‘Lasso’: Lasso linear regression (curve produced by thresholding absolute regression coefficients, whilst marker ‘X’ is single model obtained by taking only predictors with non-zero coefficients); ‘Li&Li’: penalised-likelihood approach proposed by Li and Li [21] that also incorporates network information (see main text for details); ‘corr’: absolute Pearson correlations between each protein and response. Area under the (average) ROC curve ("AUC") appears in parentheses.

We first considered two simulation models, $\gamma_{1}^{*}$ and $\gamma_{2}^{*}$, each of which is a predictor subset consisting of three proteins; PIP3, ERK1/2, p38 for Simulation 1, and RAF, MEK1/2, PKA for Simulation 2. In each case, the three proteins were chosen to be favoured by a particular prior. $\gamma_{1}^{*}$ is favoured by the intra-pathway distance prior (*f*_2_) with positive *λ*; the proteins included in $\gamma_{1}^{*}$ had a large average intra-pathway distance $\left(f_{2}(\gamma_{1}^{*})=2.5\right)$ and incorporated a medium number of pathways $\left(f_{1}(\gamma_{1}^{*})=1\right)$. $\gamma_{2}^{*}$ is favoured by either the number of pathways prior (*f*_1_) with negative *λ* or the intra-pathway distance prior (*f*_2_) with negative *λ*; the proteins included in $\gamma_{2}^{*}$ had both a small intra-pathway distance $\left(f_{2}(\gamma_{2}^{*})=0\right)$ and incorporated a small number of pathways $\left(f_{1}(\gamma_{2}^{*})=0\right)$. Since, by construction, each model is favoured by a particular prior, we can test the ability of the empirical Bayes approach to select appropriate hyperparameter values. Response data *Y * were generated using a linear model with interaction terms (4); *Y*=*A* + *BC* + *ε*, where *A,B,C *are the three influential variables.

We are especially interested in the small-sample regime that is often of interest in molecular studies. We therefore subsampled (without replacement) *n*=35 training data from the complete dataset (this matched the sample size of the drug response study reported below), and assessed predictive ability on the remaining, held-out data ( $ñ=n_{\text{tot}}-n=818$).

Subsampling was repeated to give 5,000 training/test pairs, over which results are reported below. At each iteration, only small-sample training data was used for inference. The empirical Bayes method was employed to set prior source and strength parameters (using training data only), with $\lambda \in \left[-5,5\right]$ (this specification permits a flat prior if empirical Bayes analysis supports neither prior). Posterior inclusion probabilities were then calculated as described above.

We assessed performance by comparing the true underlying model γ^*^ to the model *γ*_*τ *_obtained by thresholding posterior inclusion probabilities at level *τ*. For results from each small-sample dataset, a receiver operating characteristic (ROC) curve was constructed by plotting number of true positives $\left(\left|\gamma^{*}\cap \gamma_{\tau }\right|\right)$ against number of false positives $\left(\left|\gamma_{\tau }\setminus \gamma^{*}\right|\right)$ for varying thresholds *τ*. Figure 3a,b shows average ROC curves over the 5,000 iterations for Simulation 1 and Simulation 2, together with the area under the ROC curve (AUC). AUC is a summary of the curve and provides a measure of variable selection accuracy, with higher scores indicating better performance. The score is normalised to take a value between 0 and 1. Our Bayesian variable selection (BVS) method with empirical Bayes and linear model with interaction terms (‘BVS: EB +int’) is compared with eight other approaches: 

(i) BVS with flat prior and linear model with interaction terms (‘BVS: flat +int’);

(ii) BVS with a prior that is incorrect with respect to the true, underlying model: intra-pathway distance prior (*f*_2_) favouring small distances (*λ *= − 5) for Simulation 1 and large distances (*λ *= 5) for Simulation 2 (‘BVS: incorrect +int’);

(iii) BVS with flat prior and linear model with no interaction terms (‘BVS: flat -int’);

(iv) BVS with a Markov random field prior [19] and linear model with interaction terms (see below for further details; ‘BVS: MRF prior +int’);

(v) penalised-likelihood Lasso regression [20] using a linear model with no interaction terms (see below for further details; ‘Lasso -int’);

(vi) penalised-likelihood Lasso regression [20] using a linear model with pair-wise interaction terms (‘Lasso +int’);

(vii) a penalised-likelihood approach, proposed by Li and Li [21], based on the Lasso and also incorporates network structure information (‘Li&Li’); and

(viii) absolute correlation coefficients between each predictor and response (‘corr’).

Markov random field priors have previously been used in Bayesian variable selection to take network structure of predictors into account [19,22]. A Markov random field is an undirected graphical model *G *= (*VE*) in which vertices *V * represent variables (here, the predictors) and edges *E* represent probabilistic relationships between them. Let *A *= (*a*_*i*,*j*_) be a binary symmetric matrix with *a*_*i*,*j *_= 1 if and only if edge (*ij*)∈*E*. Then, the Markov random field prior is given by

(12) $$P(\gamma \;|\;\lambda )\propto \text{exp}\left\{\lambda \gamma^{T}\mathrm{A\gamma}\right\}.$$

The strength parameter *λ * is usually constrained to be non-negative, resulting in a prior that encourages selection of predictors whose neighbours in *G* are also included in the model. Here, we do not enforce this constraint and also allow negative values for *λ*. Negative values result in a prior that penalises models containing predictors that are neighbours in *G*. As with the proposed Bayesian variable selection method, we use a linear model with interaction terms and set *λ* with empirical Bayes. The graph structure *G* is obtained from the structure shown in Figure 2 by converting all directed edges to undirected edges.

Lasso regression performs variable selection by placing an *ℓ*_1_ penalty on the regression coefficients. This has the effect of shrinking a subset of regression coefficients to exactly zero; the predictors with non-zero coefficients are taken as the inferred model. Sparsity of the inferred model is controlled by a tuning parameter, which we set by 5-fold cross-validation. This method results in a single inferred model (i.e. point estimate). However, a full ROC curve can still be obtained by thresholding absolute regression coefficients.

The penalised-likelihood method proposed by Li and Li [21] combines a Lasso penalty with an additional penalty term that incorporates predictor network structure. Together, these penalties encourage sparse estimates for regression coefficients that are also ‘smooth’ over the network structure; that is, coefficients for neighbours in the network are encouraged to be similar. In terms of variable selection, the approach promotes models containing predictors that are neighbours in the graph. The two penalty terms each have their own tuning parameter, controlling sparsity and smoothness over the network respectively; we set these parameters by 5-fold cross-validation. (This approach, and the standard Lasso regression approach, were implemented using Matlab package glmnet[23].)

We observe that, in both simulations, the automated empirical Bayes analysis, with pathway-based priors, improves performance over the flat prior and provides substantial gains over an incorrect prior. The empirical Bayes approach selected the correct prior in 85% of iterations for Simulation 1 and 96% of iterations for Simulation 2 (for Simulation 1 correct prior parameters were *m *= 2 with *λ *> 0, median value of *λ* selected was *λ *= 3.5; for Simulation 2 correct prior parameters were *m *= 1 or *m *= 2 with *λ *< 0, median value of *λ * selected was *λ *= −5 for both *m *= 1 and *m *= 2). Since the Lasso regression method (with interaction terms) does not incorporate prior information, it is unsurprising that it is also outperformed by the empirical Bayes approach. Hence, it is fairer to compare it to Bayesian variable selection with a flat prior (and interaction terms). In Simulation 1 these regimes both show a similar performance with the Lasso approach displaying some gains at small numbers of false positives. However, in Simulation 2 the Bayesian approach offers a clear improvement in performance over Lasso regression (AUC scores of 0.91 and 0.83 respectively). Due to its inability to model combinatorial interplay, the linear model without interaction terms is outperformed by the linear model with interaction terms for both Bayesian and Lasso approaches.

In Simulation 1, the strength parameter for the Markov random field prior was set to *λ *= −5 by empirical Bayes in 86% of iterations, thereby correctly promoting models that do not contain predictors that are neighbours in the network. However, although the Markov random field prior offers improvements over a flat prior, it is outperformed by the proposed pathway-based priors at small numbers of false negatives. This is due to the fact that our intra-pathway distance prior is able to promote models with large distances between predictors, whilst the Markov random field prior can only penalise models that contain neighbours. The Markov random field prior is in general less flexible because it considers neighbours rather than distances. Intriguingly, in Simulation 2, *λ *> 0 (which correctly promotes models containing neighbours in the network) was only selected by empirical Bayes in 41% of iterations. As a result, performance of the Markov random field prior is inferior to a flat prior. We discuss this further in Discussion below.

The penalised-likelihood approach proposed in [21], incorporating network information, performs poorly in both simulations, with similar performance compared to simply looking at correlations between predictors. Whilst in Simulation 2, a clear improvement is observed over standard Lasso regression (without interaction terms), this is not the case in Simulation 1. This is because the approach promotes models containing predictors that are neighbours in the network, which reflects the true underlying model for Simulation 2 only. The general poor performance of this approach is likely due to its inability to capture combinatorial interplay since it necessarily employs a linear model without interaction terms.

Since the network-based penalised-likelihood approach [21] does not incorporate interaction terms we performed a third simulation to investigate its performance under a data-generating model without interaction terms. In particular, we used the same true underlying predictor subset as in Simulation 2 (i.e. $\gamma_{3}^{*}=\gamma_{2}^{*}$), which contains predictors that are neighbours in the network, but generated data using a linear model without interaction terms; *Y *=* A* + 2*B* + 3*C* + *ε*, where *ABC *are the three influential variables. We note that each predictor in the data-generating model has a different magnitude of influence on the response (i.e. different regression coefficients). Average ROC curves are shown in Figure 3c. Comparisons are made to other approaches as described above, but all methods now use linear models without interaction terms. As in Simulations 1 and 2 the Bayesian variable selection approach with empirical Bayes and pathway-based priors outperforms a flat prior and an incorrect prior, with empirical Bayes selecting the correct prior in 99% of iterations (correct and incorrect priors are the same as for Simulation 2). The Bayesian approach with Markov random field prior showed a similar performance to the proposed pathway-based priors (a correct value of *λ *> 0 was selected in 90% of iterations). However, the approach of Li and Li [21], whilst now more competitive compared with Simulation 2, is still outperformed by the empirical Bayes approach with pathway-based priors. Moreover, it does not display a clear improvement over Lasso regression.

**Table 1:** Synthetic response data, predictive errors from held-out test data

	**Simulation 1**		**Simulation 2**		**Simulation 3**	
	**MA**	**MAP**	**MA**	**MAP**	**MA**	**MAP**
BVS: EB prior^†^	0.819±0.004	0.850±0.004	0.837±0.004	0.889±0.005	0.899±0.002	0.918±0.002
BVS: flat prior^†^	0.845±0.004	0.919±0.005	0.845±0.004	0.919±0.006	0.904±0.002	0.927±0.003
BVS: ‘incorrect’ prior^†^	0.858±0.003	0.895±0.003	0.918±0.003	1.003±0.004	0.969±0.003	1.036±0.003
BVS: MRF prior^†^	0.830±0.004	0.877±0.005	0.871±0.004	0.920±0.006	0.886±0.002	0.911±0.002
Lasso^†^	0.791±0.003		0.790±0.003		0.913±0.002	
Li&Li	1.246±0.009		1.476±0.012		1.760±0.012	
Baseline linear	1.000±0.002		1.000±0.002		1.000±0.002	

Predictions using small-sample training data (***n *= 35**) and held-out test data (***n *= 818**; total of 5,000 train/test pairs) for Simulations 1, 2 and 3. Results shown are mean absolute predictive errors ± SEM for the following methods: Bayesian variable selection (BVS) with biologically informative pathway-based prior with source and strength parameters set by empirical Bayes, BVS with flat prior, BVS with ‘incorrect’ prior (contradicting empirical Bayes; see text for details), BVS with a Markov random field (MRF) prior, Lasso regression, penalised-likelihood approach proposed by Li and Li [21], and a baseline linear regression without interaction terms including all 11 predictors. For BVS, predictions made using the posterior predictive distribution with exact model averaging (‘MA’) and using the *maximum a posteriori* model (‘MAP’).

^‡ ^ linear model with interaction terms for Simulations 1 and 2, and without interaction terms for Simulation 3.

The failure of the incorrect prior illustrates the importance of prior elicitation. Moreover, our results demonstrate that the proposed empirical Bayes approach can select a suitable prior automatically, even under very small sample conditions (here *n *= 35). If the data is not in agreement with a proposed prior, then it is desirable that *λ *= 0 is selected by empirical Bayes, resulting in a flat prior. To test this, we used the model in Simulation 2 with a prior that favoured models with predictors from many pathways (i.e. number of pathways prior with *λ * restricted to be non-negative). This prior does not reflect the true, underlying model, which contains a small number of pathways. Empirical Bayes analysis successfully selected *λ *= 0 in 95% of iterations.

For each dataset, we used the posterior predictive distribution (Equation 10; calculated via exact model averaging) to predict responses for held-out test data. Mean absolute predictive errors, obtained by averaging over all 5,000 train/test iterations, are shown in Table 1 (‘MA’). The empirical Bayes approach with pathway-based priors shows improvements in predictive accuracy over a flat prior, and substantial improvements over both the ‘incorrect’ prior and a baseline linear model without interaction terms including all 11 predictors (i.e. no variable selection). It also outperforms the Markov random field prior in Simulations 1 and 2, and outperforms Lasso regression in Simulation 3. In Simulations 1 and 2, Lasso regression offers the best predictive performance (we note that prediction used regression coefficients obtained by maximum penalised likelihood estimation; the alternative of using Equation 11 with the single model corresponding to non-zero coefficients gave very poor predictive accuracy, inferior to the baseline linear approach; data not shown). In Simulation 3, the best predictive accuracy is provided by the Markov random field prior. The penalised regression approach proposed in [21] has very poor predictive performance across all simulations, inferior to the baseline linear model. We also found that model averaging provided gains relative to prediction using the MAP model (Equation 11), with a 5%, 7% and 3% decrease in error on average for Simulations 1-3 respectively (see Table 1, ‘MAP’).

**Figure 4:**
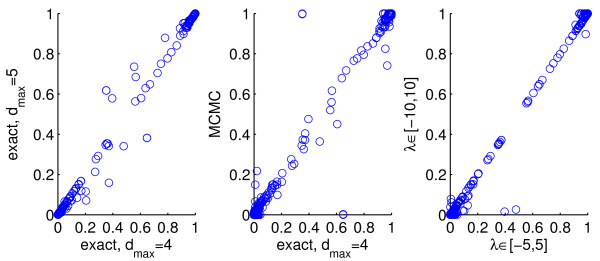
**Synthetic response data; effect of sparsity restriction and range of prior strength parameter.** Results reported in Figure 3, for the empirical Bayes approach, were obtained by exact model averaging with the number of predictors included in a model restricted to not exceed *d*_*max *_= 4. Posterior inclusion probabilities for 50 simulated datasets from Simulation 1 were compared with results obtained by exact model averaging with an increased maximum number of included predictors of *d*_*max *_= 5 (left) and using Markov chain Monte Carlo-based model averaging with no sparsity restriction (centre). Sensitivity to the range of prior strength parameter values considered by empirical Bayes was also assessed by comparing the posterior inclusion probabilities obtained with $\lambda \in \left[-5,5\right]$ to those obtained with an increased range of $\lambda \in \left[-10,10\right]$.

**Figure 5:**
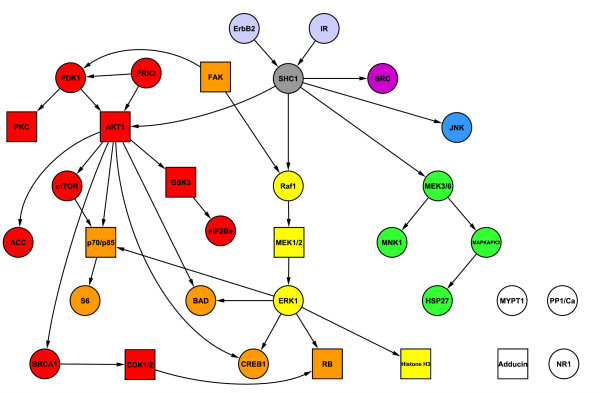
**Network and pathway structure for biologically informative priors in the cancer drug response data study.** Network constructed using information from cellsignal.com. Square nodes represent fully connected subnetworks consisting of iso-forms and phospho-forms of the named protein (see Additional file 1). Node colouring represents pathway structure. Red, blue, yellow, green and purple nodes denote 5 pathways. Orange nodes are in both the red and yellow pathways. Light grey nodes are in all 5 pathways. Dark grey node is in all pathways except the purple pathway. White nodes are not assigned to a pathway.

The only user-set parameters in the proposed method are *d*_*max*_ (the maximum number of predictors allowed in a model), and the range of values for the prior strength parameter *λ* to optimise over in empirical Bayes. We sought to check the sensitivity of our results to these parameters. As described in ‘Methods’ above, we set *d*_*max *_= 4 and considered $\lambda \in \left[-5,5\right]$. We compared the posterior inclusion probabilities inferred from 50 iterations of Simulation 1 to those obtained using (i) an increased maximum number of included predictors of *d*_*max *_= 5; (ii) Markov chain Monte Carlo-based (MCMC) inference with no restriction on number of included predictors, and (iii) an increased range for the prior strength $\lambda \in \left[-10,10\right]$ (see Figure 4). We found very close agreement in all cases, indicating that results reported do not depend on the sparsity restriction or the chosen range for *λ*.

In Simulation 2 and Simulation 3, the smallest value of *λ *= −5 was selected by empirical Bayes in a majority of iterations. The true, underlying model has the minimum possible number of pathways and intra-pathway distance. Hence, the strong (negative) prior strength is appropriate because it causes the prior to heavily penalise any model not satisfying these minima. Under the increased range for *λ*, the smallest value (*λ *= −10) was still selected in these iterations, but results were almost identical (as seen for Simulation 1 in Figure 4). This indicates that the prior was already having close to maximal influence at the lower value of *λ *= −5.

### Cancer drug response data

Aberrant signalling is heavily implicated in almost every aspect of cancer biology [24,25] and, as a result, signalling proteins are targets for many emerging cancer therapies. Here, we apply the methods proposed to probing phospho-proteomic influences on response to an anti-cancer agent Triciribine.

Phospho-protein abundance was assayed in a high-throughput manner using the KinetWorks^TM^ system (Kinexus Inc, Vancouver, Canada), for *p *= 52 proteins related to epidermal growth factor receptor (EGFR) signalling, in each of *n *= 35 breast cancer cell lines (see Additional File 1:Sections 1.1-1.2 for details). The EGFR signalling network plays a central role in breast cancer biology [26] and the cell lines used have previously been shown to retain much of the biological heterogeneity of primary tumours [27]. GI50 (log transformed) was used to quantify response to Triciribine for each of the 35 cell lines [28]. GI50 is the concentration that causes 50% growth inhibition compared to a baseline. A network (with a total of five pathways) was constructed using http://cellsignal.com (see Figure 5).

**Figure 6:**
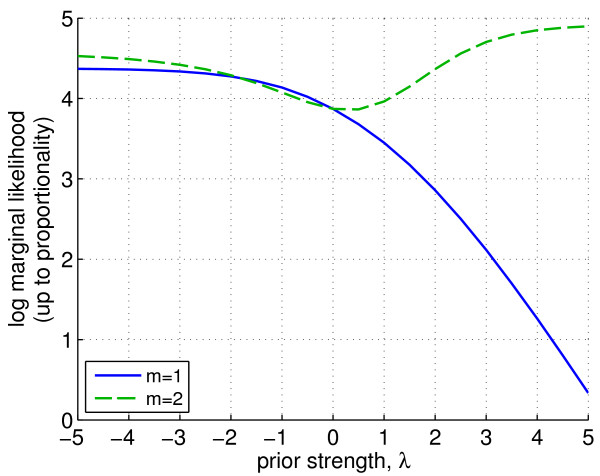
**Drug response data, empirical Bayes analysis.** Parameters controlling source of prior information and prior strength (*m* and *λ *respectively) were set objectively using the data. Log marginal likelihood (calculated exactly up to a constant) is plotted against *λ * for *m *= 1 (number of pathways prior) and *m *= 2 (intra-pathway distance prior). Parameters were set to the values with maximal marginal likelihood: *m *= 2 and *λ *= 5.

Figure 6 shows marginal likelihood scores arising from empirical Bayes. This selects the intra-pathway distance prior (*m *= 2) with hyperparameter *λ *= 5 (i.e. a prior promoting larger distances). Due to the small sample size, we tested robustness of this choice by running empirical Bayes with each data sample removed. The same prior was selected in 86% of the iterations.

**Figure 7:**
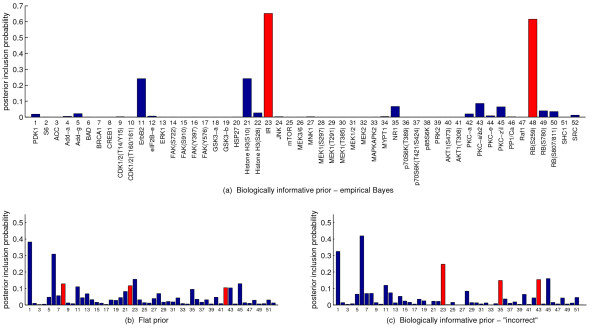
**Drug response data, posterior inclusion probabilities.** Obtained via exact model averaging with **(a)** biologically informative pathway-based model prior with parameters set objectively using empirical Bayes (*m *= 2 (intra-pathway distance), *λ *= 5 - see Figure 6), **(b)** flat prior and **(c)** "incorrect" biologically informative prior that is not optimal according to empirical Bayes analysis (*m *= 1 (number of pathways), *λ *= −5). Posterior inclusion probabilities provide a measure of how influential each protein is in determining drug response. Proteins contained in the single MAP model are shaded red.

**Table 2:** Drug response data, predictive errors from cross-validation

	**MA**	**MAP**
BVS: EB prior +int	0.84±0.12	1.00±0.16
BVS: flat prior +int	0.86±0.11	1.26±0.17
BVS: ‘incorrect’ prior +int	0.93±0.15	1.22±0.17
BVS: MRF prior +int	0.86±0.11	1.24±0.17
Lasso +int	0.73±0.10	
Li&Li	0.96±0.21	
Baseline linear	1.00±0.14	

Predictions using leave-one-out-cross-validation (see text for details). Results shown are mean absolute predictive errors ± SEM for the following methods: Bayesian variable selection (BVS) with biologically informative pathway-based prior with source and strength parameters set by empirical Bayes, BVS with flat prior, BVS with ‘incorrect’ prior (contradicting empirical Bayes; see text for details), BVS with a Markov random field (MRF) prior, Lasso regression, penalised-likelihood approach proposed by Li and Li [21], and a baseline linear regression without interaction terms including all 11 predictors. ‘+int’ denotes linear model with interaction terms. For BVS, predictions made using the posterior predictive distribution with exact model averaging (‘MA’) and using the *maximum a posteriori* model (‘MAP’).

Figure 7 shows posterior inclusion probabilities obtained under three prior regimes: empirical Bayes (intra-pathway distance prior with *λ *= 5), flat prior and an "incorrect" prior that is not optimal according to the empirical Bayes analysis (number of pathways prior with *λ *= −5). Phospho-IR and phospho-RB(S259) stand out in the empirical Bayes analysis. Triciribine targets AKT, which inhibits apoptotic processes and is heavily implicated in cancer signalling [29]. IR (insulin receptor) is a tyrosine kinase receptor, known to stimulate the AKT pathway [30], and it has been suggested that the RB/E2F pathway, which is also known to play a role in cancer [31], has an effect on AKT activity via transcriptional regulation [32]. Hence, the salience of IR and RB accords with known biology and drug mechanism. The MAP model for each prior regime is highlighted in red in Figure 7. We note that these models do not always contain the proteins with highest inclusion probabilities.

We performed Leave-One-Out-Cross-Validation (LOOCV), making predictions for the held-out test sample using both posterior model averaging (Equation 10) and the MAP model (Equation 11). The full variable selection approach, including selection of hyperparameters with empirical Bayes, was carried out at each cross-validation iteration. Table 2 shows mean absolute predictive errors, with comparisons made as in the synthetic response data study above. For the ‘incorrect’ prior, the prior source parameter not selected by empirical Bayes was used, along with the optimal strength parameter *λ * for that prior. Mirroring the synthetic data results (Simulations 1 and 2), we observe that prior elicitation with empirical Bayes provides an increase in mean predictive accuracy over a flat prior, an ‘incorrect’ prior, the Markov random field prior and the approach proposed by Li and Li [21], whilst Lasso regression has lowest mean predictive error. We note, however, that due to the very small sample size, differences in mean predictive error between these regimes are not conclusive. Yet, they all show an improvement over the baseline linear approach, and model averaging results in an average 26% decrease in predictive error over using MAP models. The prior strength parameter for the Markov random field prior was set to *λ *= −5 by empirical Bayes in every cross-validation iteration (this agrees with the selection of a pathway-based prior promoting large distances).

**Figure 8:**
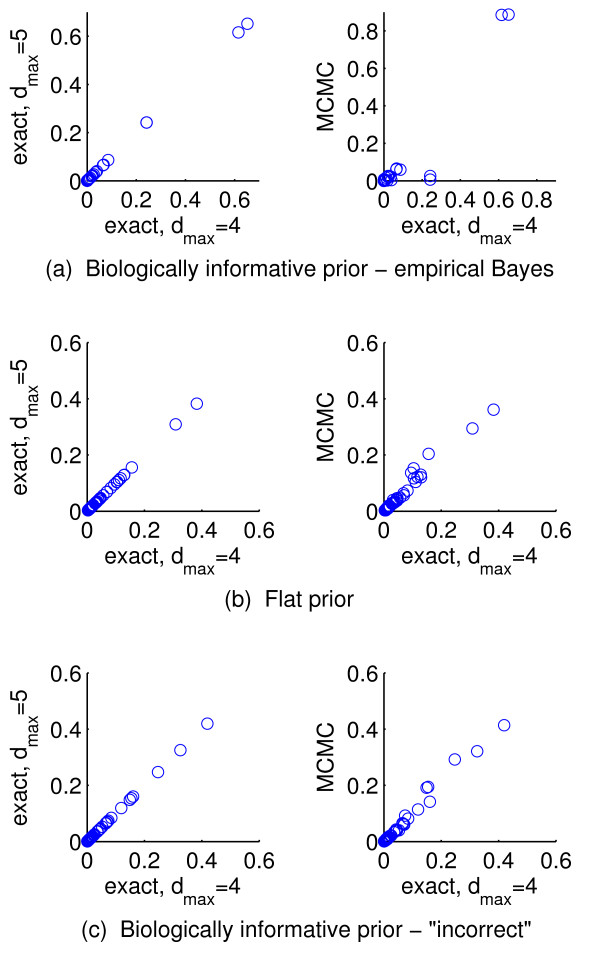
**Drug response data; effect of sparsity restriction.** Posterior inclusion probabilities in Figure 7 were obtained by exact model averaging with the number of predictors included in a model restricted to not exceed *d*_*max *_= 4. These results were compared with results obtained by exact model averaging with an increased maximum number of included predictors of *d*_*max *_= 5 (left column) and using Markov chain Monte Carlo-based model averaging with no sparsity restriction (right column).

We again checked sensitivity of results to the restriction on the number of predictors included in a model, *d*_*max *_= 4. The results in Figure 7 were compared with those obtained using an increased maximum number of included predictors of *d*_*max *_= 5 and using MCMC-based inference with no such restriction (see Figure 8). The strong agreement between *d*_*max *_= 4 and *d*_*max *_= 5 suggests that the minor differences observed between *d*_*max *_= 4 and MCMC are a result of inherent Monte Carlo error. We also see a close agreement between results in Figure 7a (using $\lambda \in \left[-5,5\right]$) and those obtained by optimising over the increased range of $\lambda \in \left[-10,10\right]$ (see Additional File 1:Figure S1). This shows that results reported do not depend on the sparsity restriction or the range of values considered for the prior strength parameter.

**Table 3:** Illustrative computation times

	**Linear model without interaction terms**					**Linear model with interaction terms**			
	***d***_***max ***_**= 2**	***d***_***max ***_**= 3**	***d***_***max ***_**= 4**	***d***_***max ***_**= 5**		***d***_***max ***_**= 2**	***d***_***max ***_**= 3**	***d***_***max ***_**= 4**	***d***_***max ***_**= 5**
p=30	0.1	1.1	8.7	9.5		0.4	4.7	38.6	374.6
p=60	0.5	10.5	114.3	−		1.8	39.4	661.6	−
p=120	2.8	116.3	−	−		8.2	350.1	−	−
p=500	150.3	−	−	−		238.7	−	−	−

Computation times (in seconds) for proposed Bayesian variable selection procedure, using empirical Bayes to select between two priors (***M *= 2**) and to set the prior strength parameter *λ * (optimisation performed over ten values of *λ*). Results shown for varying values of ***d***_***max ***_(maximum number of predictors allowed in a model) and *p* (total number of predictors), for both a linear model without interaction terms and a linear model with interaction terms. (Data and model priors generated using random numbers and results are averages over three iterations. Computation performed on a standard single-core personal computer; 1.6GHz, 2GB RAM. ‘-’ denotes a (***p***,***d***_***max***_) regime where the procedure failed due to insufficient memory).

## Discussion

Model priors incorporating biological information can play an important role in variable selection, especially at the small sample sizes characteristic of molecular studies. In applications where there are multiple sources of prior information, or multiple possible prior specifications, the empirical Bayes approach we put forward permits objective selection and weighting. This aids prior elicitation and guards against the use of mis-specified priors. We demonstrated that a biologically informative prior, with hyperparameters set by empirical Bayes, can have benefits over both a flat prior and a subjectively formed prior which is incorrect with respect to the underlying system. We also observed that, whilst Lasso regression can offer some improvement in predictive performance over the Bayesian approaches, its accuracy in selecting the correct underlying model (i.e. variable selection) can be inferior to the proposed empirical Bayes approach, thereby affecting interpretability of results. Empirical Bayes approaches have previously been used in variable selection, but with standard Bernoulli-distributed priors [33,34].

We developed informative priors in the context of protein signalling based on two high-level features derived from network information: the number of pathways a subset of predictors incorporates and the intra-pathway distance between proteins in a proposed model. This formulation used the entire network structure in an intuitive way, removing the the need to specify individual prior probabilities for each variable and avoiding assumptions of prior independence between variables.

Our pathway-based priors form part of a growing literature on exploiting existing domain knowledge to aid inference, especially in the small sample setting. For example, recent variable selection studies also make use of graph structure within a Bayesian Markov random field prior [19,35,36] and within a non-Bayesian framework [21,37,38], essentially preferring models containing predictors that are neighbours in the graph. This is similar in spirit to the special case of our prior where the network consists of a single pathway and short intra-pathway distances are strongly preferred.

We compared our pathway-based priors to the Markov random field prior, but found in Simulation 2 that empirical Bayes frequently set the prior strength parameter to an incorrect value, resulting in a prior that penalises models containing predictors that are neighbours in the network, instead of promoting them. This is likely due to the parameterisation of the Markov random field prior, which is not agnostic to the number of included predictors in the model |γ|; addition of a predictor to a model could lead to a substantial increase in the prior score. Indeed, it has previously been noted that Markov random field priors can be unstable with the occurance of phase transitions in |γ| [19]. Hence, the prior prefers less sparse models, but these models do not agree well with the data, as more complex models are penalised by the marginal likelihood. In contrast, our distance prior is based on an average distance measure and so is somewhat indifferent to |γ|. In Simulation 3, we do not observe this behaviour of the Markov random field prior; since the linear model does not include interaction terms, the model complexity does not increase as sharply with |γ| and so there is less disagreement between the prior and the marginal likelihood. We note that biologically informative priors have also been used for classification [22,39,40] and network inference [41-43].

We also compared our approach to the network-based penalised-likelihood method proposed by Li and Li [21]. It performed poorly in Simulations 1 and 2, primarily due to its inability to capture nonlinear interplay. However, even in Simulation 3, with no interaction terms in the data-generating model, it failed to match the performance of our proposed empirical Bayes approach with pathway-based priors. This could be due to lack of similarity between the regression coefficients in the data-generating model, which goes against an assumption of the penalised-likelihood approach; that coefficients are similar for predictors that are neighbours in the network. This could also explain its poor predictive performance.

We used a continuous regression framework with interaction terms. Whilst discrete models are naturally capable of capturing non-linear interplay between components, the discretisation process results in a loss of information. Continuous models avoid this loss, but the response is usually assumed to depend linearly on predictors. The product terms in our model provide the possibility of capturing influences on the response of interest by interplay between predictors, including higher-order interactions. Chipman [44] and Jensen *et al.*[3] have employed a related approach allowing pairwise interactions only. We note that, under our formulation, model complexity grows rapidly with number of included predictors. However, complex models are naturally penalised by the marginal likelihood formulation giving overall sparse, parsimonious models, yet allowing for complex interplay via product terms.

We carried out variable selection using exact model averaging. This was made possible by means of a sparsity restriction. Sparsity constraints have been employed in previous work in Bayesian variable selection [4,45] and also in the related setting of inference of gene regulatory networks [42,46]. The sparsity-constrained approach proposed is attractive as it yields exact posterior probabilities and facilitates exact empirical Bayes analysis. Sparsity is a reasonable assumption in settings where it is likely that only a few predictors play a key role in influencing a response. In such settings, and where data is of small-to-moderate dimensionality, our exact approach is fast and deterministic with no requirement of MCMC convergence diagnostics. This, together with empirical Bayes and the choice of parameter priors, results in the overall approach having very few user-set parameters.

In applications of higher dimensionality, where the exact calculation is no longer feasible, empirical Bayes can still be performed using an approximate conditional marginal ‘likelihood’ approach as seen in George and Foster [33] and Yuan and Lin [34]. This involves optimisation over the model space instead of averaging. MCMC, with the selected hyperparameter values, can then be used to estimate inclusion probabilities. Alternatively, a fully Bayes MCMC approach could be taken, which places a prior on the hyperparameters and integrates them out (see e.g. [14]).

Illustrative computational times for our approach are shown in Table 3, for four values of *p* (number of predictors) and four values of *d*_*max*_(maximum number of predictors allowed in a model). We also considered linear models with and without interaction terms. Empirical Bayes was used to select between two priors (*M *= 2) and to set the prior strength parameter (optimisation performed over ten values of *λ*). The computation time scales as $d_{\text{max}}p^{d_{\text{max}}}$ for the model without interaction terms and $(2^{d_{\text{max}}}-1)p^{d_{\text{max}}}$ for the model with interaction terms. We see that the approach is fast on datasets of moderate dimensionality (∼100 variables) with *d*_*max *_= 3. We note that shortage of memory was the limiting factor on our machine. Computational time could also be improved by using multiple cores to calculate empirical Bayes marginal likelihood scores for multiple values of *λ*simultaneously.

We showed examples of automated selection between multiple sources of ancillary information, but, rather than selecting a single source, the methods proposed could be generalised to allow combinations of complementary information sources as seen in Jensen *et al.*[3]. Whilst our priors were based on pathway and network structure, the methods can also permit integration and weighting of publicly available data, which while plentiful, can be of uncertain relevance to a given study.

## Conclusions

In this paper we have proposed an empirical Bayes method for objective selection and weighting of biologically informative prior information for integration within Bayesian variable selection. The method is computationally efficient, exact and has very few user-set parameters. We developed informative pathway-based priors in the context of protein signalling and illustrated our method on synthetic repsonse data. We demonstrated that in situations where there are several plausible formulations for the prior, it is capable of selecting the most appropriate. In particular, the approach has potential to significantly improve results by guarding against mis-specification of priors. Comparisons were made to alternative methods, demonstrating that the proposed approach offers a competitive variable selection performance. We have also shown an application on cancer drug response data and obtained biologically plausible results. Our method is general and can be applied in any setting with multiple sources of prior knowledge.

## Competing interests

The authors declare that they have no competing interests.

## Authors contributions

SMH designed the priors, carried out all computational analyses and wrote the paper. SM conceived the study, provided feedback on all aspects and revised the manuscript. RMN and NB carried out the cell line assays. WLK and SZ carried out the drug response assays. PTS and JWG led the biological aspects of the work and provided feedback. All authors read and approved the final manuscript.

## Supplementary Material

Addtional file 1 **Cancer drug response application.** Tables of proteins and cell lines included in the analysis, further details of the experimental procedure and Figure S1. Click here for file
