# Supplementary material for: Integrating biological knowledge into variable selection: an empirical Bayes approach with an application in cancer biology
Source: BMC Bioinformatics. 2012 May 11;13:94. doi: 10.1186/1471-2105-13-94 (PMC3503557; doi:10.1186/1471-2105-13-94)
Supplement: Addtional file 1 — Cancer drug response application. Tables of proteins and cell lines included in the analysis, further details of the experimental procedure and Figure S1. [file 1471-2105-13-94-S1.pdf]

Additional File 1

Integrating biological knowledge into variable selection: an empirical Bayes approach with an application to cancer biology

Steven M. Hill [1,2,3], Richard M. Neve [4], Nora Bayani [5], Wen-Lin Kuo [5], Safiyyah Ziyad [5], Paul T. Spellman [6], Joe W. Gray [6] and Sach Mukherjee [1,3,2]

[1]The Netherlands Cancer Institute, 1066 CX Amsterdam, The Netherlands, [2]Centre for Complexity Science and [3]Department of Statistics, University of Warwick, Coventry CV4 7AL, U.K., [4]Genentech Inc., San Francisco, CA 94080, [5]Life Sciences Division, Lawrence Berkeley National Laboratory, Berkeley, CA 94720, [6]Center for Spatial Systems Biomedicine, Oregon Health & Science University, Portland, OR 97239

1 Cancer drug response data

1.1 Proteins and cell lines

1	PDK1 (S244)	19	GSK3-β (S9)	37	p70S6K-α (T421/S424)
2	S6 (S235)	20	HSP27 (S15)	38	p85S6K-β (T444/S447)
3	ACC (S80)	21	Histone H3 (S10)	39	PRK2 (T816)
4	Adducin-α (S726)	22	Histone H3 (S28)	40	AKT1 (S473)
5	Adducin-γ (S693)	23	IR (Y999)	41	AKT1 (T308) (S729)
6	BAD (S99)	24	JNK (T183/Y185)	42	PKC-α (S657)
7	BRCA1 (S1497)	25	mTOR (S2448)	43	PKC-$\alpha/\beta 2$ (T638/T641)
8	CREB1 (S133)	26	MEK3/6 (S189/S207)	44	PKC-ϵ
9	CDK1/2 (T14/Y15)	27	MNK1 (T209/T214)	45	PKC-ζ/ι (T410/T403)
10	CDK1/2 (T161/Y160)	28	MEK1 (S297)	46	PP1/Ca (T320)
11	ErbB2 (Y1248)	29	MEK1 (T291)	47	Raf1 (S259)
12	eIF2B-ϵ (S540)	30	MEK1 (T385)	48	RB (S259)
13	Erk1 (T202/Y204)	31	MEK1/2 (S217/S221)	49	RB (S780)
14	FAK (S722)	32	MEK2 (T394)	50	RB (S807/S811)
15	FAK (S910)	33	MAPKAPK2 (T222)	51	SHC1 (Y349/Y350)
16	FAK (Y397)	34	MYPT1 (T696)	52	SRC (Y529)
17	FAK (Y576)	35	NR1 (S896)		
18	GSK3-α (S21)	36	p70S6K-α (T389)		

Table S1: Phospho-proteins involved in our cancer drug response study.

1	600MPE	13	HCC38	25	MDAMB436
2	AU-565	14	HCC70	26	SK-BR-3
3	BT-20	15	Hs587T	27	SUM149PT
4	BT-474	16	LY2	28	SUM159PT
5	BT-483	17	MCF10A	29	SUM185PT
6	BT-549	18	MCF12A	30	SUM225CWN
7	CAMA-1	19	MCF7	31	SUM52PE
8	HCC1143	20	MDAMB134VII	32	T-47D
9	HCC1187	21	MDMB157	33	UACC-893
10	HCC1500	22	MDAMB175	34	ZR-75-1
11	HCC1569	23	MDAMB231	35	ZR-75-8
12	HCC202	24	MDAMB361		

Table S2: Breast cancer cell lines involved in our cancer drug response study.

1.2 Proteomics

Cell lysates: For preparation of protein lysates cells were grown to 60-80% confluency in appropriate media (Neve *et al.*, 2006). Cultures were placed on ice, media aspirated and washed in ice cold PBS containing 1mM phenylmethylsulfonyl fluoride (PMSF) and then with a buffer containing 50mM HEPES (pH7.5), 150mM NaCl, 25mM b-glycerophsphate, 25mM NaF, 5mM EGTA, 1mM EDTA, 15mM pyrophosphate, 2mM sodium orthovanadate, 10mM sodium molybdate, leupeptin (10mg/ml), aprotinin (10mg/ml) and 1mM PMSF. Cells were extracted in the same buffer containing 1%Nonidet-P40. Lysates were then clarified by centrifugation and frozen at $-80\,^{\circ}\mathrm{C}$. Protein concentrations were determined using the Bio-Rad protein assay kit. Phopshoproteome analysis was performed at Kinexus (http://www.kinexus.ca/) on their $\mathrm{KinetWorks}^{\mathrm{TM}}$ platform.

1.3 Sensitivity to range of prior strength parameter λ

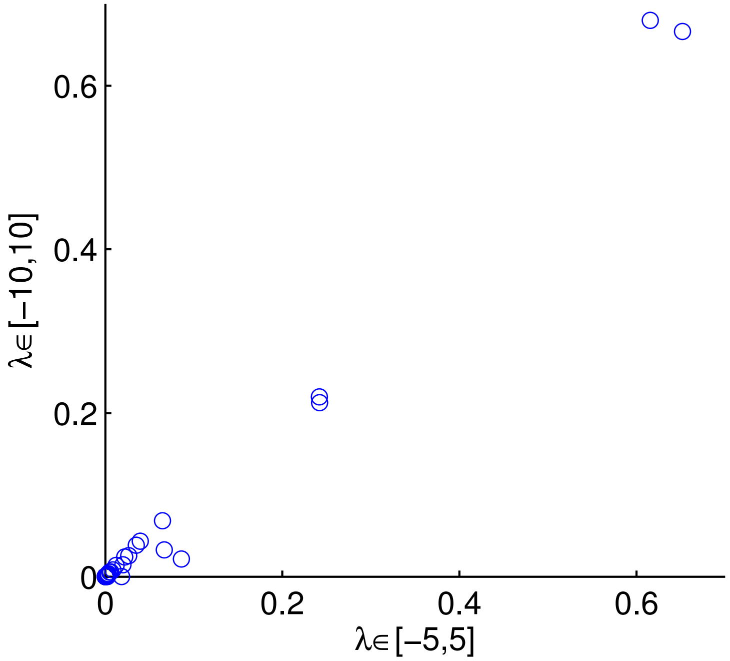

Figure S1: Drug response data; sensitivity to range of prior strength parameter. Posterior inclusion probabilities reported in Figure 7a (Main Text) were obtained using the proposed empirical Bayes approach, with prior strength parameter optimised over the interval $\lambda \in [-5, 5]$. These results are compared to those obtained with an increased range of $\lambda \in [-10, 10]$.

References

Neve, R. M. *et al.* (2006). A collection of breast cancer cell lines for the study of functionally distinct cancer subtypes. *Cancer Cell*, **10**, 515–527.
